# *Notes from the Field*: Increase in New Delhi Metallo-β-Lactamase–Producing Carbapenem-Resistant Enterobacterales — New York City, 2019–2024

**DOI:** 10.15585/mmwr.mm7423a2

**Published:** 2025-06-26

**Authors:** Katelynn Devinney, Nicole Burton, Karen A. Alroy, Addie Crawley, Cherry-Ann Da Costa-Carter, Molly M. Kratz, Ying Lin, Jorge Montfort-Gardeazabal, Thomas Portier, Celina Santiago, Ulrike Siemetzki-Kapoor, Matthew Sullivan, Rain J. Wiegartner, Tristan D. McPherson, William G. Greendyke

**Affiliations:** 1 New York City Department of Health and Mental Hygiene, New York, New York.

**Summary**

**What is already known about this topic?**

Enterobacterales, a large group of gram-negative bacteria, can acquire resistance to broad-spectrum carbapenem antibiotics through mechanisms that include production of enzymes (carbapenemases). Although the most common carbapenemase in the United States is *Klebsiella pneumoniae* carbapenemase (KPC), the incidence of New Delhi metallo-β-lactamase (NDM) has been increasing.

**What is added by this report?**

In New York City, the annual number of NDM-positive carbapenem-resistant Enterobacterales (CRE) cases increased from 2019 (58 cases) to 2024 (388). In 2024, the number of NDM-positive CRE cases surpassed KPC-positive CRE cases.

**What are the implications for public health practice?**

In New York City, NDM has become the most common carbapenemase. Infections with KPC- and NDM-positive CRE might require different antibiotics. Providers should be aware of predominant carbapenemases within their clinical settings when initiating antibiotic treatment for patients with CRE infections.

Enterobacterales comprise a large group of gram-negative bacteria, including *Escherichia coli* and *Klebsiella* species; infections with these organisms often require treatment with a class of broad-spectrum antibiotics known as carbapenems. Numerous mechanisms can result in the emergence of carbapenem-resistant Enterobacterales (CRE), including the production of enzymes (carbapenemases) that render the antibiotics ineffective in killing bacteria. CRE cause health care–associated infections resulting in substantial morbidity and mortality; carbapenemase-producing CRE are particularly concerning because carbapenemase genes are easily spread via plasmid-mediated genetic elements.* *Klebsiella pneumoniae* carbapenemase (KPC) has been the predominant carbapenemase among Enterobacterales in the United States since 1996 (*1*). Another carbapenemase, New Delhi metallo-β-lactamase (NDM), is less common in the United States, confers resistance to antimicrobials commonly used to treat KPC-positive CRE (e.g., the combination antibiotic ceftazidime-avibactam) (*2*), and has previously been associated with returning international travelers (*3*). The prevalence of NDM-positive CRE has increased in New York City (NYC) health care settings, including long-term care facilities (LTCFs) (*4*). The NYC Health Department observed a notable and sustained increase in NDM-positive CRE cases from 2019 to 2024, indicating that local treatment recommendations might need to be modified in response to changing carbapenemase epidemiology. This report describes trends in carbapenemase epidemiology in NYC during 2019–2024.

## Investigation and Outcomes

### Data Source

In 2018, the NYC Health Code was amended to mandate that laboratories electronically report CRE among NYC residents to the NYC Health Department, including organism identification, antimicrobial susceptibility testing and, when available, carbapenemase test results.^†^ In NYC, carbapenemase testing (including for KPC, NDM, oxacillinases, imipenemase, and Verona integron–encoded metallo-β-lactamase)^§^ is increasingly performed internally by clinical laboratories; public health laboratories establish isolate submission protocols for clinical laboratories that do not have carbapenemase testing capabilities and routinely conduct carbapenemase testing on CRE isolates.^¶^ To evaluate trends in carbapenemase epidemiology, the frequency of detected carbapenemases among CRE cases (clinical specimens collected from NYC residents that tested positive for *E. coli*, *K*. *pneumoniae*, *Klebsiella aerogenes*, *Klebsiella oxytoca*, or *Enterobacter cloacae* and were carbapenem-resistant or tested positive for a carbapenemase)** diagnosed during 2019–2024 was determined, and patient and isolate characteristics were described. This activity was designated nonhuman subjects research (i.e., public health surveillance) by the NYC Health Department and did not require institutional review board assessment.

During 2019–2024, among 7,114 CRE cases reported in NYC, 3,293 (46%) had accompanying carbapenemase test results, with proportions ranging from 40% (444 of 1,105) in 2021 to 54% (751 of 1,378) in 2024. From 2019 to 2024, the number of NDM-positive CRE cases increased from 58 to 388, while the number of CRE cases with KPC-positive results remained relatively stable (range = 277–332 annually). In 2024, NDM surpassed KPC as the most frequently reported carbapenemase among CRE cases (Figure). Based on patient’s street address at the time of diagnosis, 30% of 1,069 NDM-positive CRE cases occurred among residents of LTCFs^††^; this proportion peaked at 38% (36 of 96) in 2021 and was 25% (96 of 388) in 2024. During 2019–2024, age-adjusted NDM-positive CRE incidences^§§^ increased annually citywide, from less than one per 100,000 residents in 2018–2021 to 1.5 in 2022, 2.9 in 2023, and 3.8 in 2024. Among NDM-positive CRE cases, *K. pneumoniae* was the most frequently reported organism (68%); urine was the most common initial specimen source (43%) (Supplementary Table).

**FIGURE:**
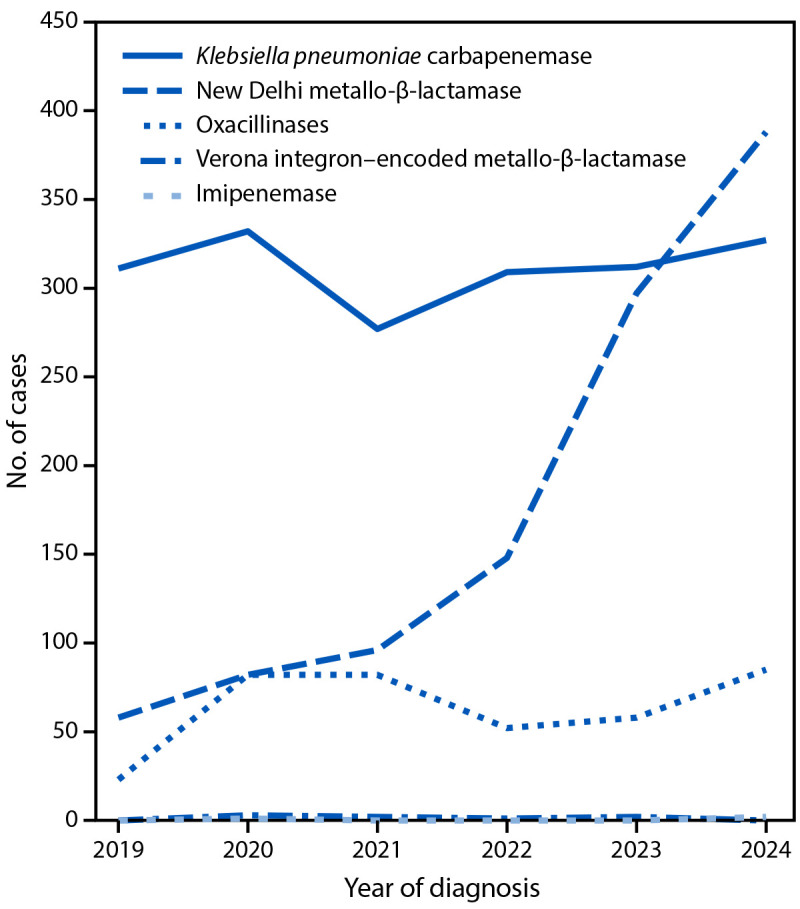
Number of carbapenem-resistant Enterobacterales cases with a detected carbapenemase,* by carbapenemase type^†^ and diagnosis year — New York City, 2019–2024 **Abbreviations: **CRE = carbapenem-resistant Enterobacterales; NYC = New York City. * Among 7,114 CRE cases reported in NYC, 3,293 (46%) had carbapenemase results, with proportions ranging from 40% (444 of 1,105) in 2021 to 54% (751 of 1,378) in 2024. ^†^ Verona integron–encoded metallo-β-lactamase was detected in one case in 2020 and two cases in 2024. Imipenemase was detected in three cases in 2020, two in 2021, one in 2022, and two in 2023.

## Preliminary Conclusions and Actions

The reasons for the increase in NDM-positive CRE since 2019 are not fully understood. The increase in the proportion of NDM-positive CRE among persons without known LTCF exposure suggests the possibility of community transmission beyond health care settings where CRE transmission has previously been identified (*4*,*5*). However, ascertainment of the settings where these infections were acquired was not possible with the available disease data. Although incomplete carbapenemase testing and reporting, possibly due to limited resources and complex reporting requirements, contributed to incomplete ascertainment of NDM-positive CRE incidence in NYC, the relatively stable prevalence of other reported carbapenemases suggests that the increase in NDM incidence is not solely attributable to increased carbapenemase testing and reporting by clinical laboratories.

Given the reported increase of NDM, providers treating NYC residents at risk for CRE could consider empiric therapy effective against NDM-producing CRE, such as cefiderocol or ceftazidime/avibactam plus aztreonam, particularly for patients with severe CRE infection, and modify therapy as needed based on carbapenemase or susceptibility test results.^¶¶^ Other jurisdictions should be aware of the potential for similar increases in NDM-positive CRE cases. The NYC Health Department is implementing prospective whole genome sequencing of CRE isolates to improve CRE cluster detection, including NDM-positive CRE, and better understand local transmission.

## Footnotes

*
Antibiotic Resistance Threats in the United States 2019 | CDC

†
NYC Department of Health: Reportable Diseases and Conditions: Article 11

§ Carbapenemase testing includes molecular and phenotypic methods, indicating presence of a carbapenemase gene or the production of a carbapenemase enzyme, in accordance with the 2023 Council of State and Territorial Epidemiologists (CSTE) carbapenemase-producing organism case definition. National Notifiable Diseases Surveillance System Carbapenemase-producing organisms, screening. 2023 Case Definition | CDC

¶ Clinical laboratories are not required to submit isolates to public health laboratories for carbapenemase testing; public health laboratories coordinate with clinical laboratories to establish isolate submission protocols for laboratories that are unable to conduct carbapenemase testing.

** If multiple organisms and at least one carbapenemase were detected in a single specimen, then each organism was counted separately and considered to harbor all detected carbapenemases. Clinical specimens collected from the same person that tested positive for *E. coli*, *K*. *pneumoniae*, *K. aerogenes*, *K. oxytoca*, or *E. cloacae* and that had unique organism-carbapenemase combinations were counted separately, and a single such case was only counted once during the study period, in alignment with the 2023 CSTE carbapenemase-producing organisms case definition.

†† LTCF residency status was determined by matching the patient’s street address at the time of report to a geocoded list of known facilities and by searching for selected key words (e.g., nursing home) in the patient’s address. Detailed information regarding where the infection occurred or for cases not in LTCFs was not available.

§§ Incidences were calculated by county of residence and diagnosis year, using intercensal population estimates updated in 2022 (for 2019) and 2024 (for 2020–2024) with a base file from the 2020 U.S. Census Bureau standard population. Patients were grouped into eight age categories (0–17, 18–24, 25–34, 35–44, 45–54, 55–64, 65–74, and ≥75 years), and age-adjusted rates were calculated using direct standardization and weighting to the U.S. Census Bureau 2000 standard population.

¶¶
IDSA 2024 Guidance on the Treatment of Antimicrobial Resistant Gram-Negative Infections
